# Visualization of airborne droplets generated with dental handpieces and verification of the efficacy of high-volume evacuators: an in vitro study

**DOI:** 10.1186/s12903-023-03725-1

**Published:** 2023-12-07

**Authors:** Min Jung Kim, Mana Kuroda, Yoshikazu Kobayashi, Takahisa Yamamoto, Takako Aizawa, Koji Satoh

**Affiliations:** 1 Department of Dentistry and Oral-Maxillofacial Surgery, Fujita Health University, School of Medicine, 1-98 Dengakugakubo, Kutsukake, Toyoake, Aichi 470-1192 Japan; 2 Department of Mechanical Engineering, National Institute of Technology, Gifu College, 2236-2 Kamimakuwa, Motosu-city, Gifu, 501-0495 Japan

**Keywords:** Coronavirus disease 2019, Aerosol-generating procedure, High-volume evacuator, Dental rotary instrument

## Abstract

**Background:**

The COVID-19 pandemic led to concerns about the potential airborne transmission of the virus during dental procedures, but evidence of actual transmission in clinical settings was lacking. This study aimed to observe the behavior of dental sprays generated from dental rotary handpieces and to evaluate the effectiveness of high-volume evacuators (HVEs) using laser light sheets and water-sensitive papers.

**Methods:**

A dental manikin and jaw model were mounted in a dental treatment unit. Mock cutting procedures were performed on an artificial tooth on the maxillary left central incisor using an air turbine, a contra-angle electric micromotor (EM), and a 1:5 speed-up contra-angle EM (×5EM). Intraoral vacuum and extraoral vacuum (EOV) were used to verify the effectiveness of the HVEs. The dynamics and dispersal range of the dental sprays were visualized using a laser light sheet. In addition, environmental surface pollution was monitored three-dimensionally using water-sensitive papers.

**Results:**

Although the HVEs were effective in both the tests, the use of EOV alone increased vertical dispersal and pollution.

**Conclusions:**

The use of various types of HVEs to reduce the exposure of operators and assistants to dental sprays when using dental rotary cutting instruments is beneficial. The study findings will be helpful in the event of a future pandemic caused by an emerging or re-emerging infectious disease.

**Supplementary Information:**

The online version contains supplementary material available at 10.1186/s12903-023-03725-1.

## Background

The novel coronavirus disease 2019 (COVID-19), which originated in December 2019, has caused one of the most severe pandemics in history. According to the World Health Organization (WHO), in March 2020, severe acute respiratory syndrome coronavirus 2 (SARS-CoV-2) was transmitted between humans via respiratory droplets and contact routes [1]. Simultaneously, the WHO mentioned that airborne transmission of coronavirus might be possible in specific circumstances and settings wherein procedures that generate aerosols are performed. Therefore, in August 2020, the WHO recommended that healthcare professionals involved in dental practice should postpone treatment, except for that in urgent cases, to prevent cross-infection from dental treatment during the COVID-19 pandemic [2]. Indeed, high viral loads of SARS-CoV-2 have been detected in the oral fluids of coronavirus-positive [3, 4] and asymptomatic patients [5, 6]. However, the spread of COVID-19 associated with dental treatment is yet to be reported in actual clinical settings.

Aerosol is defined as a “sol in which the dispersed phase is a solid, a liquid, or a mixture of both, and the continuous phase is a gas” [7]. Aerosol-generating procedures (AGPs) are generally performed in oral healthcare settings worldwide. An AGP is defined as any medical, dental, and patient care procedure that produces airborne particles of < 5 μm in size (aerosols), which can remain suspended in the air, travel over a distance, and may cause infection if inhaled [2]. Droplets larger than aerosols are defined as splatter, droplets, or droplet nuclei, and are commonly referred to as dental sprays together with aerosols [8]. There are four main sources of dental sprays: air-water syringes, ultrasonic instruments, high-speed turbines, and lasers [8]. Electric micromotors (EMs) can also generate airborne droplets [9]. The spread and dynamics of dental sprays have been previously studied using microbiological methods [10–15], tracer dyes [16–21], particle counters [22, 23], and other bioaerosol sensors [24]. Microbiological techniques involve collecting and culturing microorganisms from samples such as dental personnel’s clothing, environmental surfaces, and the air as well as observing their spread. Tracer dye methods incorporate dye in the coolant of rotary handpieces. Particle counters and bioaerosol sensors operate by detecting scattered light when a laser beam or light-induced fluorescence interacts with particulate matter as it flows through the instrument’s flow path. While these methods offer precise quantification, they cannot directly visualize the dynamics of dental sprays. In addition, attempts have been made to visualize the dynamics of sprays (including aerosols and splatters) during dental procedures [9, 25–29]. These studies used high-speed imaging and broadband or monochromatic laser light-sheet illumination to experimentally visualize dental sprays produced by an ultrasonic scaler. In this regard, high-volume evacuators (HVEs) can help in reducing aerosol dispersion during dental procedures. However, to the best of our knowledge, there have been no studies that have visualized the dynamics of the dental sprays and verified the effectiveness of these suction devices while using dental rotary handpieces.

This study aimed to examine the behavior of dental sprays generated from dental rotary handpieces using qualitative and quantitative experiments (i.e., laser light sheets and water-sensitive paper tests) that allow direct and indirect visualization of the dynamics of sprays and to validate the efficacy of HVEs. We hypothesized that the use of HVEs would reduce dental sprays and that the behavior would be different for air turbines (ATs) using compressed air and EMs.

## Methods

This in vitro experiment was conducted to simulate a clinical environment of the outpatient dentistry service at our institutional hospital. The treatment rooms were separated by walls without windows from ceiling to floor, the back side of the dental unit was open to provide passage for staff, and mechanical ventilation was provided once every 30 min through the supply and exhaust vents in the ceiling. All experimenters wore safety glasses and were not allowed to stand facing the laser light source.

### Instrumental setup

**Fig. 1:**
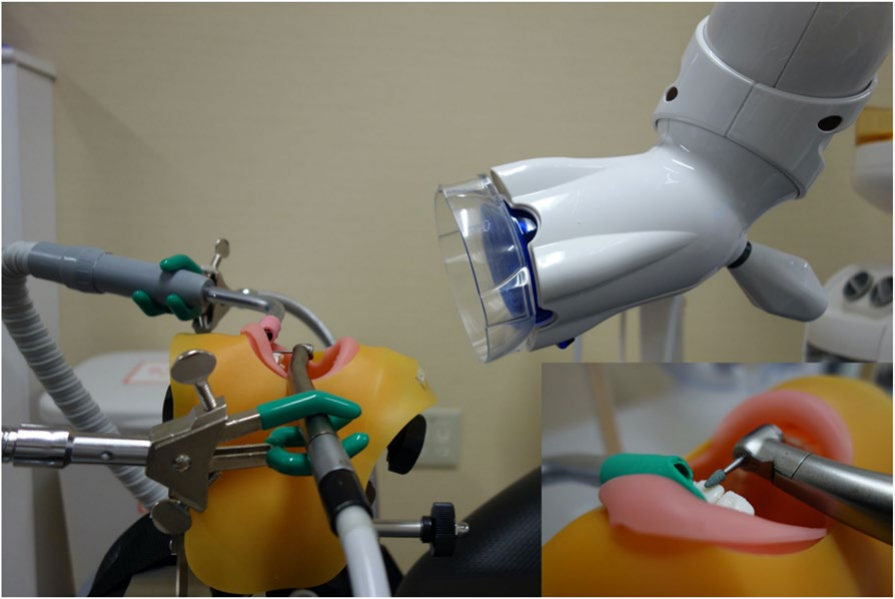
The dental manikin and jaw model settings in a dental treatment unit. High-speed handpieces (air turbine, contra-angle electric micromotor, and 1:5 speed-up contra-angle electric micromotor) were operated to simulate cutting procedures

A dental manikin (Simple Manikin III; Nissin Dental Products, Kyoto, Japan) and jaw model (Hard Gingiva Jaw Model, Nissin Dental Products) were mounted in a dental treatment unit (Signo G50; Morita, Saitama, Japan) in the horizontal position. An AT (TwinPower Turbine P PAR-EX-O-DI, Morita; 450,000 rpm), a contra-angle EM (TorqTech CA-DC-O, Morita; 40,000 rpm), and a 1:5 speed-up contra-angle EM (×5EM; TorqTech CA-5IF-O, Morita; 200,000 rpm) were used to simulate cutting procedures on an artificial tooth on the maxillary left central incisor (Fig. 1). The water injection volume per minute for the AT and EM was measured three times in advance using a measuring cylinder, and the average water injection volume was determined to be 50.5 mL/min and 43.0 mL/min, respectively. A flexible arm stand was used to secure the handpieces, such that the bur was parallel to the tooth axis. To verify the effectiveness of the HVEs, an intraoral vacuum (IOV) accompanying the dental treatment unit and an extraoral vacuum (EOV) (Free arm Arteo-T, Tokyo Giken Inc., Japan) was used. The IOV was grasped with a flexible arm stand or hand such that it was positioned on the labial side of the artificial tooth. This placement was selected to ensure that the direction of spray dispersal matched that of water injection from the instruments, as determined in preliminary experiments. Moreover, based on these preliminary experiment findings, dental sprays were rarely observed outside the manikin’s mouth during procedures involving the molars. As a result, both qualitative and quantitative evaluations were considered difficult, and the molars were excluded from the experiments. The EOV was placed 10 cm from the mouth, as recommended by the manufacturer. The operating conditions of the HVEs were set as follows: (1) no HVE, (2) EOV only, (3) IOV only, and (4) EOV + IOV.

### Visualization and analysis of dental sprays with laser light sheet

**Fig. 2:**
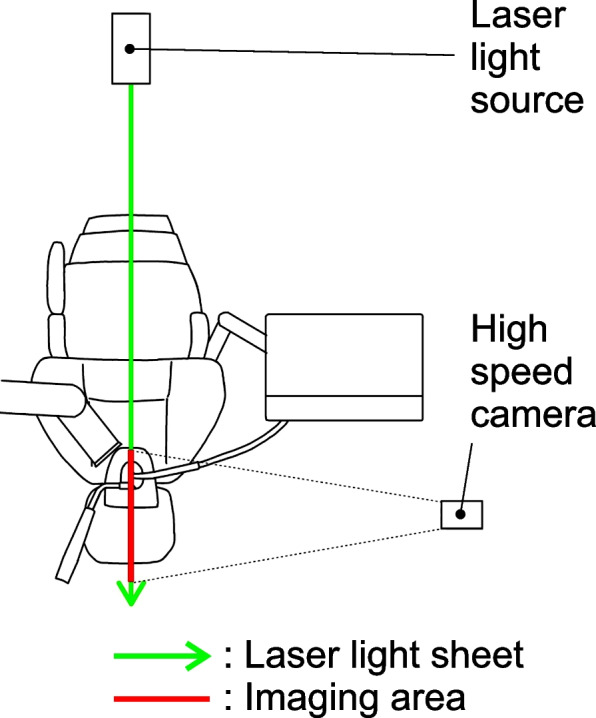
Equipment settings for dental spray visualization with laser light sheet

**Fig. 3:**
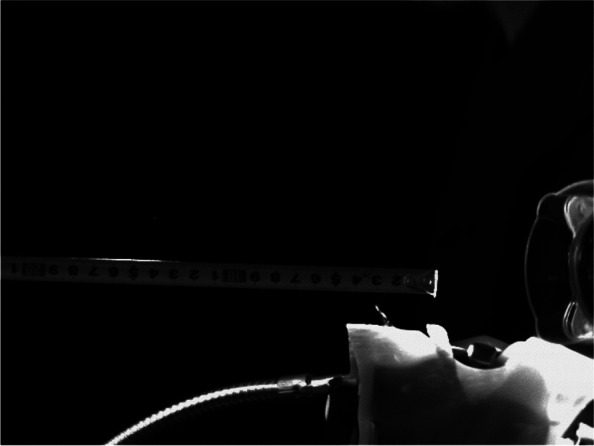
Images taken by the high-speed camera (calibration data). Each image was taken with an angle of view of 640 × 480 pixels, and the actual size of one pixel was 0.45868 mm

To evaluate dental spray dispersion, a laser light sheet (PIV Laser G2000, Katokoken Co., Kanagawa, Japan; 532 nm wavelength, 2 W output power, continuous wave) was used for visualization. A high-speed camera (k5, Katokoken Co.) was used to capture images (Fig. 2). The frame rate was 1000 frames per second. The imaging angle of view was 294 × 220 mm, and its resolution was 640 × 480 pixels (Fig. 3). The dynamics and dispersal range of the visualized dental sprays were qualitatively evaluated from the chin of the manikin to the position of the surgeon. Note that the actual size of one pixel that can be captured at this angle of view is 458.68 μm, which is larger than the WHO-defined boundary between droplets and droplet nuclei (5 μm); therefore, this experiment is only an observation of droplets. For quantitative analysis, five still images were randomly captured from the video taken in each operating condition. The images of the experimenter and manikin were blacked out in the monochrome still images, and the ratio of the white droplets occupying the screen was calculated and averaged. We used an image analysis software (ImageJ version 1.53 t; National Institutes of Health, Bethesda, MD) to binarize all image data with the same luminance threshold.

### Evaluation of environmental surface pollution with water-sensitive papers

**Fig. 4:**
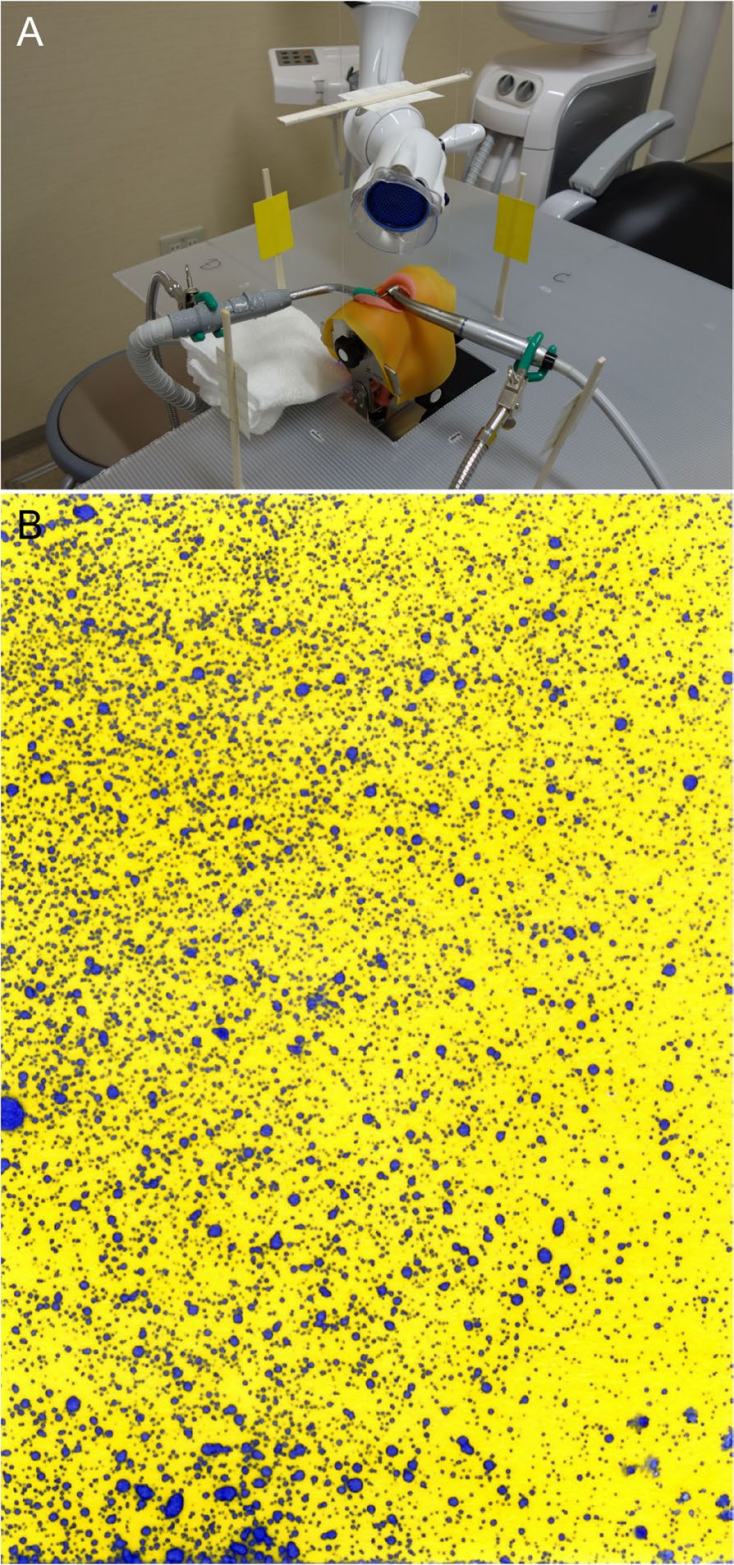
Experimental setup. **A** Setting up an experiment to observe the pollution of environmental surfaces using water-sensitive paper. **B** Scanned image of water-sensitive paper (12 o’clock direction, 10-cm distance, 1:5 speed-up contra-angle electric micromotor). The area where water adheres to the surface turns blue

To evaluate the pollution of environmental surfaces in clinical dental settings, we observed three-dimensional spray dispersal with water-sensitive papers (WSPs; Syngenta Water-Sensitive Paper, 52 × 76 mm; Spraying Systems Co., Glendale Heights, IL). WSPs were fixed at equal intervals in the 12, 3, 6, and 9 o’clock positions around the manikin’s head using plastic boards and disposable chopsticks. For vertical measurements, a fishing line was hung from the ceiling, to which a WSP was attached. Three 30-s mock treatments were performed under conditions wherein the WSPs were placed at distances of 10, 20, and 30 cm from the manikin’s head (Fig. 4). We used ImageJ to binarize all image data with the same luminance threshold, and the area discolored by water adhesion was measured and averaged over three times.

### Statistical analysis

Comparisons between each operating condition were made using one-way analysis of variance, followed by post-hoc group comparisons using Tukey’s honestly significant difference test. All statistical analyses were performed using JMP®13 (SAS Institute Inc., Cary, NC, USA), and the statistical significance level was set at α = 0.05.

## Results

### Visualization and analysis of dental sprays with laser light sheets

**Fig. 5:**
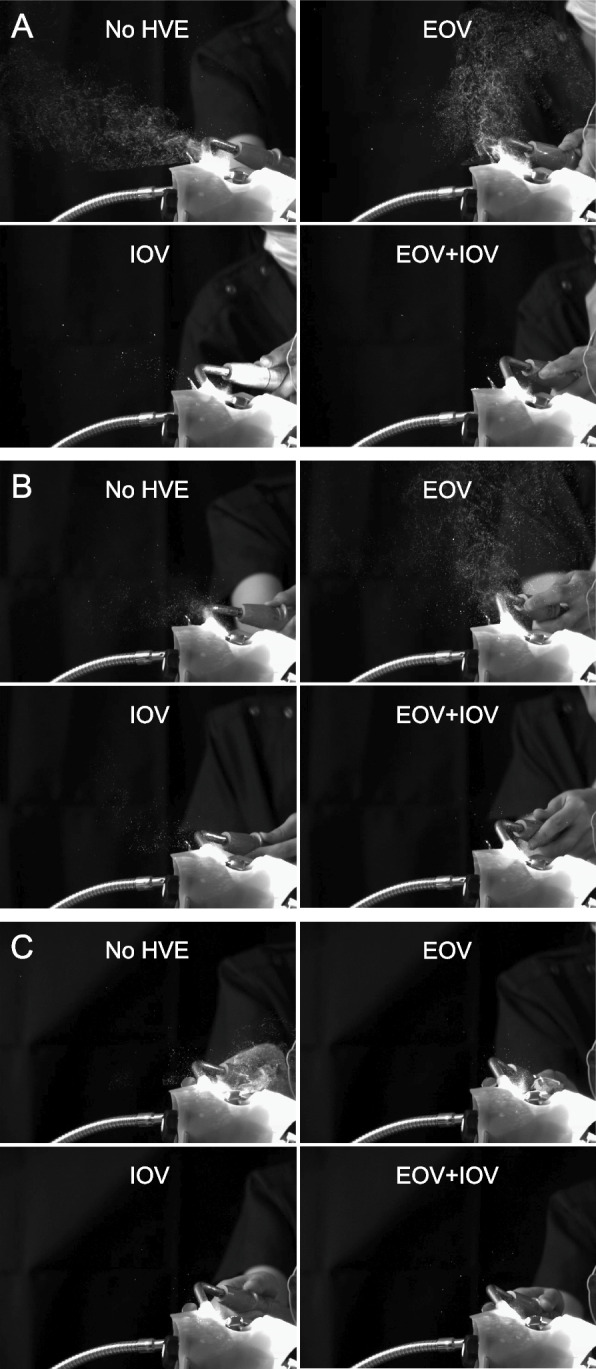
Captured images taken by the high-speed camera under each condition. **A** air turbine, **B** contra-angle electric micromotor, and **C** 1:5 speed-up contra-angle electric micromotor. HVE, high-volume evacuator; IOV, intraoral vacuum; EOV, extraoral vacuum

**Fig. 6:**
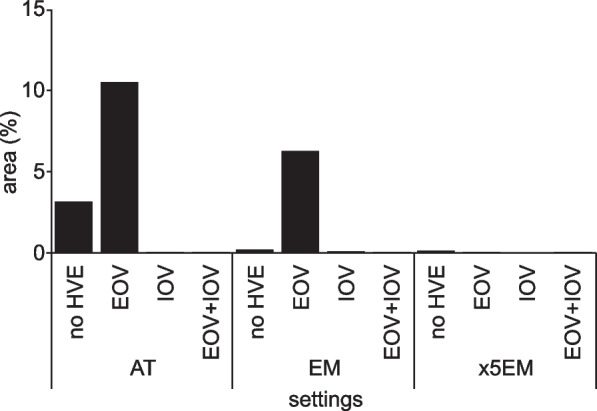
Results of quantitative analysis of laser light sheet test. The area occupied by droplets on the screen was in the order of air turbine (AT), contra-angle electric micromotor (EM), and 1:5 speed-up contra-angle electric micromotor (×5EM). With AT and EM, the area occupied by droplets on the screen was greater in the EOV only condition than in the no HVE condition. HVE, high-volume evacuator; IOV, intraoral vacuum; EOV, extraoral vacuum

Without HVEs, the AT generated the greatest amount of dental spray dispersal (Fig. 5). The AT and EM exhibited more extensive spray dispersal in the 12 o’clock direction. In contrast, dispersal of the ×5EM was more noticeable in the 6 o’clock direction. When only an EOV was used, the amount of dental spray dispersed in the 12 o’clock direction decreased for all handpieces. After dispersing extensively in the vertical direction, the dental sprays moved toward the 6 o’clock direction where the EOV was placed. When only an IOV was used, the amount of dispersal was reduced for all handpieces, with only a few large droplets dispersed in the 12 o’clock direction. When an EOV was used, the amount of dispersal was further reduced, and large droplets were almost unnoticeable. Additional movie files show this in more detail (see Additional files 1, 2 and 3). Quantitative analysis showed that the area occupied by droplets on the screen was in the order of AT, EM, and ×5EM. With AT (*p* < 0.0001) and EM (*p* < 0.0001), the area occupied by droplets on the screen was greater in the EOV only condition than in the no HVE condition (Fig. 6, Supplementary data sheet).

### Evaluation of environmental surface pollution with WSPs

**Fig. 7:**
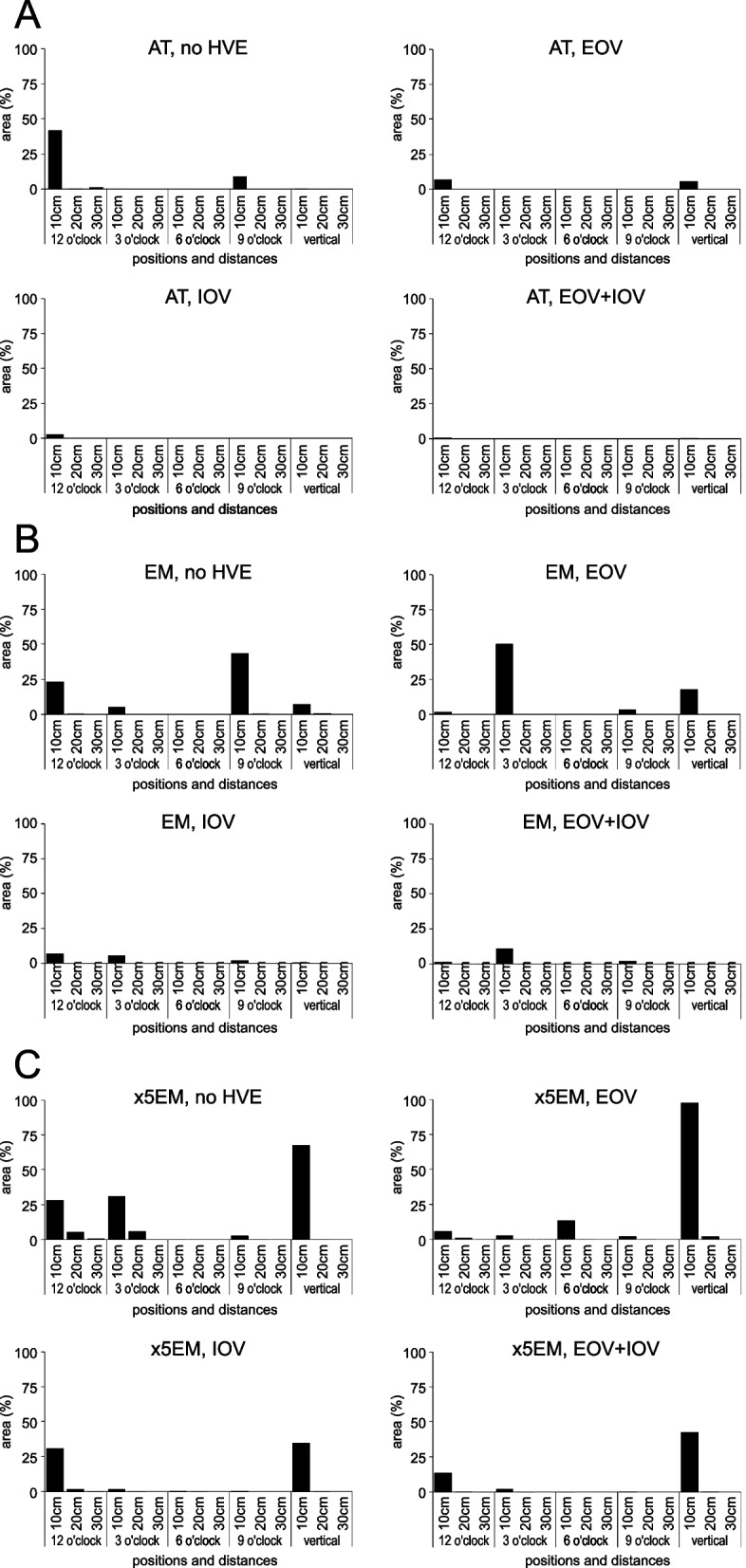
Results of water-sensitive paper tests. **A** air turbine, **B** contra-angle electric micromotor, and **C** 1:5 speed-up contra-angle electric micromotor. At distances greater than 20 cm from the handpieces, spray contamination was less than 10% of the water-sensitive area in all examinations. Although high-volume evacuators were effective, contamination tended to increase in the direction of the extraoral vacuum when it was used alone

The stained area at the 20- and 30-cm positions was less than 1% for AT and EM; however, ×5EM had larger stained areas at some of the 20-cm positions (1.64–5.90%) but significantly lesser than those at the 10-cm positions (*p* < 0.001). Therefore, the results are summarized below for the staining area at the 10-cm positions (Fig. 7, Additional file 4).

When no HVE was used: AT showed staining at 12 o’clock (41.9%) and 9 o’clock (8.96%); EM had staining at 9 o’clock (43.5%), 12 o’clock (23.3%), vertical (7.21%), and 3 o’clock (5.48%) directions; while ×5EM showed staining in the vertical direction (67.7%), at 3 o’clock (31.0%), 12 o’clock (28.1%), and 9 o’clock (2.81%). When only EOV was used: AT showed significantly increased staining in the vertical direction (from 0.53 to 5.78%, *p* < 0.001). EM had a significant increase in staining at 3 o’clock (50.41%) and in the vertical direction (17.88%), (*p* < 0.001). ×5EM had a significant increase in staining at 6 o’clock (from 0.01 to 13.58%) and the vertical direction (97.59%), (*p* < 0.001). When only IOV was used, the amount of stained area decreased in all directions for AT and EM. For ×5EM, the stained area decreased in all directions except at 12 o’clock. When EOV and IOV were used together, there was a significant decrease in the stained area in all directions with one exception: EM at the 3 o’clock direction, where no significant change was observed (*p* = 0.99). As for ×5EM, using both EOV and IOV led to a slight increase in the stained area in the vertical direction compared to using IOV alone, but this increase was not statistically significant (*p* = 0.68).

## Discussion

In the present study, the AT generated the highest dental spray dispersal as measured using a laser light sheet. An AT is a cutting instrument that rotates a bar at high speed by injecting compressed air into the rotor blades. In contrast, the EM uses a small motor inside the handpiece to rotate the bar. Both instruments use water injection to cool the heat generated during cutting. An AT produces atomized sprays because compressed air fractures the cooling water.

By contrast, EMs produce relatively large-sized particles because they do not use compressed air. Atomized sprays tend to scatter owing to their small particle sizes [8]. Sergis et al. reported that 1:5 speed-increasing handsets could be used without atomization or ejection of high-velocity droplets when specific operating parameters (80,000–100,000 rpm) are selected [9]. However, cutting of enamel, dentin, and restorative materials using these parameters is inefficient, due to the low velocity, rendering it an unrealistic setting in clinical practice [9].

An IOV is generally used to suction the spray generated during cutting and the pooled oral liquid. In this experiment, there was almost no difference between the use of both EOV and IOV and the use of an IOV alone, suggesting that an IOV alone can significantly reduce spray dispersion. This result supports the findings of a previous report [25]. The direction of spray dispersal matched that of water injection from the instruments. Therefore, placing an IOV opposite to the water injection is most effective.

An EOV is commonly used to suction cutting debris generated when grinding dentures and other materials at the chair side. In this study, when only the EOV was used, the vertical dispersal of droplets increased, suggesting the possibility of increased exposure to the surgeon. This was thought to be caused by the suction effect of the EOV placed opposite of the direction of dispersal of droplets ejected from the handpieces. However, when the EOV and IOV were used together, the number of droplets with large particle sizes decreased compared with that when only the IOV was used, suggesting an accumulation effect, albeit limited.

Spray splatter was observed with WSP to assess environmental surface contamination in a clinical dental setting. Compared to that at the 10-cm position, spray contact on the WSP was minimal at the 20- and 30-cm positions. However, the use of the suction device significantly reduced water detection, even at these positions, indicating that it also reduced environmental surface pollution. In particular, the decrease in spray contact in the 12 o’clock direction was similar to the results of the visualization experiment using the laser light sheet. The increase in vertical detection when using the ×5EM, EOV alone, and both EOV and IOV was thought to be due to water injection without using pressurized air. In other words, the increased vertical detection was thought to be the effect of slow-velocity droplets rolling up by the EOV. However, the EM using the same mechanism did not produce similar results.

One limitation of this study is that we did not observe aerosols. Traditionally, droplets have been defined as being > 5 μm in size; droplet nuclei (i.e., particles arising from desiccation of suspended droplets) have been associated with airborne transmission and are defined as < 5 μm in size [30, 31]. For SARS-CoV-2, although the main routes for the spread of infection are droplets and contact, aerosols can be vectors in specific environments [32]. Meanwhile, the smallest droplet size that could be observed at the angle of view in this study was approximately 500 μm. Although the scattered light captured droplets with a particle size smaller than this, aerosols < 5 μm were not visualized directly. If a small angle of view is used, the droplet nuclei can be observed; however, it is impossible to observe the dynamics over a wide area, such as exposure to the dental practitioner or distribution throughout the environment. In addition, epidemiological studies have demonstrated that the ventilation of the entire environment, such as opening windows, entrance screening (waterfront measures), and using N95 masks only when treating positive patients or those strongly suspected of being infected are sufficiently effective in preventing SARS-CoV-2 infection [33]. In any case, the patient’s saliva should be considerably diluted by cooling water from dental rotary cutting instruments, and horizontal transmission of SARS-CoV-2 is unlikely to occur.

## Conclusions

The findings of this study indicate that the use of various types of HVEs reduces the exposure of operators and assistants to dental sprays when using dental rotary cutting instruments is beneficial. However, using EOV requires caution because of the potential for low-velocity droplets to travel vertically. Additionally, we confirmed that wearing personal protective equipment (PPE) and cleaning the environment after the procedure are essential, given the considerable risk of pollution of environmental surfaces, practitioners’ bodies, and clothing surfaces after treatment. However, it is yet to be verified as to how many viruses and bacteria are present in droplets during dental procedures; therefore, further microbiological studies are needed.

In the event of a future pandemic caused by an emerging or re-emerging infectious disease, policymakers may be able to determine the appropriate requirements for PPE in dental care and the scale of practice restrictions by investigating the transmission routes of pathogenic microorganisms at an early stage. We believe this study will be beneficial in this regard.

### Supplementary Information

**Additional file 1.** Visualization of dental sprays with laser light sheets for air turbine.

**Additional file 2.** Visualization of dental sprays with laser light sheets for electric micromotor.

**Additional file 3.** Visualization of dental sprays with laser light sheets for 1:5 speed-up contra-angle electric micromotor.

**Additional file 4.** Raw dataset of the analyses.

## Data Availability

All data generated or analyzed during this study are included in this published article [and its supplementary information files].

## Abbreviations

COVID-19: Coronavirus disease 2019
WHO: World Health Organization
SARS-CoV-2: Severe acute respiratory syndrome coronavirus 2
AGP: Aerosol-generating procedure
EM: Electric micromotor
HVE: High-volume evacuator
AT: Air turbine
×5EM: 1:5 speed-up contra-angle electric micromotor
IOV: Intraoral vacuum
EOV: Extraoral vacuum
WSP: Water-sensitive paper
PPE: Personal protective equipment
